# Current issues and areas for improvement in the Korean Dental Hygienist National Licensing Examination: an expert Delphi survey among dental hygienists

**DOI:** 10.3352/jeehp.2017.14.21

**Published:** 2017-09-13

**Authors:** Yoon-Sook Hwang, Hyun-Sook Kang, Soo-Hwa Kim, Hee-Jung Moon, Sun-Mi Lee, Jae-Yeon Jung, Su-Jeong Hwang, Jung-Eun Ha

**Affiliations:** 1 Department of Dental Hygiene, Hanyang Women's University, Seoul, Korea; 2 Department of Dental Hygiene, Suwon Women's University, Suwon, Korea; 3 Department of Dental Hygiene, Yeoju Institute of Technology, Yeoju, Korea; 4 Department of Dental Hygiene, Dongnam Health University, Suwon, Korea; 5 Department of Dental Hygiene, College Of Medical Science, Konyang University, Daejeon, Korea; 6 Department of Dental Hygiene, Division of Health Science, Baekseok University, Cheonan, Korea; Hallym University, Korea

**Keywords:** Dental hygienists, Dental health surveys, Dental scaling, Republic of Korea

## Abstract

**Purpose:**

This study aimed to investigate current issues and areas for improvement in the Korean Dental Hygienist National Licensing Examination (KDHNLE) through an expert Delphi survey.

**Methods:**

A Delphi survey was conducted from May through August 2016 in Korea. This Delphi survey included 20 persons representing the field of dental hygiene (7 groups from various dental hygiene-related organizations). The Delphi survey was administered through e-mail as 3 rounds of questionnaire surveys regarding the issues facing the KDHNLE and potential solutions to those challenges. The primary Delphi survey was an open questionnaire. In each round, subjects’ responses were categorized according to the detailed themes of their responses. The minimum value of the content validity ratio of the survey results was determined by the number of panels participating in the Delphi survey.

**Results:**

Issues facing the KDHNLE were identified from the results of the Delphi survey. The following 4 items had an average importance score of 4.0 or higher and were considered as important by over 85% of the panels: the failure of the practical test to reflect actual clinical settings, the focus of the practical test on dental scaling, the gap between the items evaluated on the national examination and actual practical work, and insufficiency in strengthening the expertise of licensed dental hygienists. The following items were suggested for improvement: more rigorous rater training, adjustment of the difficulty of the licensing examination, the introduction of a specialized dental hygienist system, and more rigorous refresher training for licensed dental hygienists.

**Conclusion:**

Based on the above results, the KDHNLE should be improved according to the core competencies of dental hygienists, including on-site clinical practice experience.

## Introduction

Dental hygienists are professionals who serve to promote oral health in the community through oral health education, preventive dental care, and dental health consultation and management [1]. The purpose of the Korea Dental Hygienist National Licensing Examination (KDHNLE) is to determine the qualifications of dental hygienists who seek licensure to practice dental hygiene. In the United States and Canada, these national examinations assess the ability to understand important information from the basic biomedical, dental, and dental hygiene sciences, and the ability to apply such information in an issue-solving context. Practical tests are conducted with the particular goal of verifying techniques of dental hygiene care to be applied in dental health clinics [2,3]. However, in Korea, rote-type written tests have generally been provided, with the practical test only evaluating the ability of examinees to operate simple instruments; such an examination might be insufficient to evaluate the practical abilities and issue-solving skills of dental hygienists [4]. While Korea’s improved economic status has prolonged the average human life span, disease structures have also changed due to the aging popuation and the burden of various chronic diseases [5]. Furthermore, dental healthcare has been shifting its focus from treatment to preventive care. As the demand for dental services related to periodontal diseases and systemic diseases has increased, diverse strategies have become necessary to improve the performance of dental hygienists [6]. Therefore, this study aimed at identifying the current issues facing the KDHNLE, because the competence of dental hygienists must be assessed in accordance with changes in the healthcare environment.

## Methods

### Study design

A Delphi survey was conducted.

### Subjects

The Delphi survey included 8 representatives from dental hygiene-related organizations, 2 dental hygienists from public health centers, 2 industrial dental hygienists, 2 university hospital-affiliated dental hygienists, 2 dental clinic dental hygienists, 2 dentists, and 2 dental hygiene professors, for a total of 20 persons.

### Questionnaire

The Delphi survey was administered through e-mail as 3 rounds of questionnaire surveys. The surveys were conducted on May 11, July 7, and August 8, 2016, respectively. The primary survey was an open questionnaire that allowed experts to freely write their opinions (Appendix 1). The secondary survey was a structured questionnaire, which categorized the detailed reasons presented by 20 experts in the primary survey. Specifically, opinions were divided into categories in accordance with the study’s purpose and were then quantified using a 5-point Likert scale. The final survey showed the average, the standard deviation, and the quadrants of the results of the secondary survey, thereby showing the general extent of agreement in the experts’ opinion. Then, the response of each individual subject was displayed for comparison with the responses of the entire panel. The last round of surveys had the potential to show the same results as the secondary round, or to differ depending on the opinions of the panel. Additionally, minority opinions of the panel were important in the final questionnaire. If a response was out of the interquartile range, the subject was asked to write a brief justification to explain the discrepancy.

### Data analysis

The minimum value of the content validity ratio (CVR) of the survey results was determined by the number of panels participating in the Delphi survey. That is, only the items with a CVR value equal to or higher than the minimum value at the significance level of 0.05 may be considered valid. Thus, the minimum CVR value was set to be 0.42 for the 20 panels. The criterion for the degree of convergence, representing the convergence of the responses from the Delphi survey, was set to be 0.5 or lower, and the criterion for the degree of consensus, representing the consensus among the respondents, was set to be 0.75 or higher. The criterion for rating consistency, which is important in a survey of experts to determine the acceptability of the responses, was set to be 80% or higher.

The final Delphi survey was performed by excluding the questions that showed a low CVR in the secondary survey. Specifically, these were 1 question about the issues facing the KDHNLE system and 3 questions about the solutions to the issues of the KDHNLE system. The essential factors identified from the results of the secondary Delphi survey were rated using a 5-point Likert scale, and the rating results were statistically analyzed and compared with the results of the secondary Delphi survey.

### Ethical approval

The study was conducted in compliance with the principles of the Helsinki Declaration. Ethical clearance of the study was granted by the Institutional Review Board of Hanyang Women’s University (IRB no. AN01-201605-HR-007-01) after receiving informed consent from the subjects.

## Results

The issues facing the KDHNLE identified from the primary survey, which incorporated open-ended questions, are as follow: failure of the practical test to reflect actual clinical settings; focus of the practical test on dental scaling; inefficiency of the time and cost spent on the practical test; deviation among raters on the practical test; gap between the items evaluated on the national examination and actual practical work; inappropriateness of the ratios of questions for individual work types; lack of subjects related to fundamental knowledge; difficulties in evaluating in-depth knowledge; lack of discrimination capacity of the examination; need to develop new questions for the written examination; and insufficiency in strengthening the expertise of licensed dental hygienists.

The analysis of the CVR of the individual items related to problems in the secondary and final surveys is shown in Table 1. Raw data were available from Supplement 1. In the final survey, the items with an average importance score of 4.0 or higher were as follows, in order: failure of the practical test to reflect actual clinical settings” (average score= 4.35), insufficiency in strengthening the expertise of licensed dental hygienists (average score= 4.35), gap between the items evaluated on the national examination and actual practical work (average score= 4.30), and focus of the practical test on dental scaling (average score= 4.20).

The solutions to the issues facing the KDHNLE system identified from the results of the primary survey with open-ended questions are as follows: introduction of an all-time practical test system; conversion of the direct practical test to an indirect practical test; more rigorous rater training; integration of the licensing examination into a written examination; modification of the question development criteria; adjustment of the number of questions among subjects; increase in the total number of questions; introduction of problemsolving questions; addition of a written examination regarding fundamental subjects; adjustment of the difficulty of the national examination; introduction of a specialized dental hygienist system; and more rigorous refresher training for licensed dental hygienists.

The analysis of the CVR of the individual items related to solutions to the issues facing the KDHNLE in the secondary and final surveys is shown in Table 2. The items with an average importance score of 4.0 or higher were introduction of a specialized dental hygienist system (average score= 4.40), more rigorous refresher training for licensed dental hygienists (average score= 4.30), more rigorous rater training (average score= 4.30), introduction of problem-solving questions (average score=4.10), and adjustment of the difficulty of the national examination (average score= 4.05) in the final survey.

## Discussion

This Delphi survey was conducted to identify the problems facing the current KDHNLE system, solutions to those problems, and strategies for improvement. Our results showed that the main problems of the KDHNLE system were failure of the written and practical tests to reflect actual clinical settings and insufficiency in the follow-up management of licensed dental hygienists.

The current written examination of the KDHNLE only evaluates an individual’s intellectual capabilities, especially simple recall capacity, and is therefore insufficient to evaluate practical work capabilities and problem-solving competence [7]. The proportion of rote questions was reduced from 67.5% in 2006 to 36.5% in 2014, and the proportions of data presentation and problem-solving questions were increased from 25.0% and 5.7% in 2006 to 50.0% and 13.5% in 2014, respectively [8,9]. However, the written exam of KDHNLE does not reflect actual clinical settings, since the data presentation and problem-solving questions were only based on the content of dental hygiene textbooks. Nam et al. [10] reported that a dental calculus detection and removal test using a manikin (with only 2 molars) during 4 minutes was also problematic for assessing the minimum capacity required for dental hygienists. The practical examination is focused on the evaluation of simple instrument-manipulating capacity for a short time (only 4 minutes), and is thus insufficient to evaluate the practical work capabilities and problem-solving competence required from dental hygienists in actual clinical settings. Thus, it is necessary to improve the national examination to evaluate the overall practical capabilities of dental hygienists.

The following improvements related to the KDHNLE may be suggested. First, it is essential to organize the core competencies of dental hygienists in a way that is associated with tasks. Second, based on the core competencies, questions in the written examination and new content areas in the practical skill test are needed to develop competency requirements as part of a long-term policy initiative. In addition, the content areas of the examination should be integrated, and questions for evaluating competence-centered problem-solving capabilities should be developed. Moreover, the practical examination should include practical questions that may evaluate not only dental plaque detection and removal, but also extended work areas. Third, short-term policies related to the practical examination are suggested to improve the fairness of the test. The examination should be offered 2 or 3 times a year, not just once a year, to provide more opportunities for the applicants. Since it may be difficult to achieve an objective rating if 2 or more examinations are provided in a year, the education for the raters should be made more rigorous with the goal of ensuring objective ratings.

In North America, where the field of dental hygiene first developed, almost all aspects of dental hygiene education and licensing programs are defined and regulated [11,12]. The Commission on Dental Accreditation has developed standardized guidelines to be used in educational institutions, administrative departments, and examinations for dental hygienists [11]. The educational content and examination system of the curriculum focus on competence in a dental hygienist’s practical skill sets. As a specific example, the time for practice sessions is regulated to develop skills that can be applied in actual fieldwork. Likewise, various standards are established in detail, and universities adhere to these standards. On July 2016, the Korean Dental Hygienists Association proposed a set of core competencies of dental hygienists [12]. In the future, based on these criteria, it will be necessary to develop a standard curriculum and examination that can improve the performance of dental hygienists. Furthermore, while the most effective way to improve dental hygienists’ competency is on-site clinical practice, a lack of on-site clinical practice sites and standard guidelines is apparent. The quality of on-the-job training can be improved by means of efforts on the part of the Korean Dental Hygienists Association to develop supplementary educational programs and to improve the quality of practical education. In addition, it is necessary to establish standard guidelines that apply to the different needs of each training environment.

The main issue related to the follow-up system identified in the Delphi survey was insufficiency in strengthening the expertise of licensed dental hygienists. Recently, it has become necessary for comprehensive problem-solving skills to receive more emphasis than basic knowledge in performing practical tasks in dental clinics as a dental hygienist. Even though examinees earn their ability to perform dental hygiene work through qualifications such as a national examination, they may not be able to perform their tasks sufficiently in a rapidly-changing environment. Therefore, in several countries, a follow-up management system is carried out through education in the form of re-certification systems [13]. In the United States, a dental hygiene licensing renewal system (every 2 to 3 years) started in 1969 in Minnesota, expanded to Wyoming in 2006, and currently operates in 49 states [14]. Moreover, in Canada, dental hygienists must participate in educational courses to maintain their license. The United Kingdom provides a refresher course, but it is not obligatory. Australia pays for the next year of dental hygiene licensing, and Japan does not manage licensed dental hygienists [13]. In Korea, dental hygienists who do not take a refresher course are not allowed to work in the clinic according to the Medical Technician Act. Since the structure of the refresher course system may be vulnerable, an improved system is needed for the continued training of dental hygienists. Therefore, based on the core competencies of dental hygienists, refresher education should be conducted considering the work and employment situations of dental hygienists, such as clinics and various employment agencies.

Only the opinions of dental hygienists were analyzed, because our Delphi survey was not conducted among experts in various fields. Moreover, the subjects, who were selected from 7 groups representing dental hygienists, were not distributed in equal proportions. Thus, a limitation could arise from the inability to explore issues facing the KDHNLE and areas for improvement from other perspectives. However, we thought that dental health experts would be able to propose concrete and specific ideas for improving the KDHNLE. In the future, a Delphi survey including experts from various fields and further quantitative research will be needed to confirm these policy proposals regarding the KDHNLE.

In order to promote these policies, organic cooperation among the Korean Dental Hygienists Association, Korea Dental Hygiene Education Evaluation Institute, Korea Health Personnel Licensing Examination Institute, and other related ministries and organizations will be needed. Since the national examination is implemented to evaluate an individual’s work capabilities as a dental hygienist, with appropriate professional knowledge and skills, the examination system should be regularly reviewed and improved to foster the training of competent dental hygienists required by society.

## Figures and Tables

**Table 1.** Issues facing the Korean Dental Hygienist National Licensing Examination identified from the Delphi survey

Issue	Secondary survey		Final survey	
	CVR	Mean士SD	CVR	Mean士SD
Failure of the practical test to reflect actual clinical settings	0.90	4.40±0.75	0.90	4.35±0.74
Focus of the practical test on dental scaling	0.70	4.10±0.78	0.90	4.20±0.69
Deviation among raters on the practical test	0.50	3.95±0.83	0.60	3.90±0.55
Gap between the items evaluated on the national examination and actual practical work	0.80	4.30±0.66	0.90	4.30±0.57
Lack of discrimination capacity of the examination	0.30	3.80±0.83	0.30	3.75±0.63
Need to developing new question for the written examination	0.80	3.95±0.76	0.80	3.95±0.60
Insufficiency in strengthening the expertise of licensed dental hygienists	0.80	4.15±0.59	0.90	4.35±0.58

CVR, content validity ratio; SD, standard deviation.

**Table 2.** Improvements to address the issues facing the Korean Dental Hygienist National Licensing Examination identified from the Delphi survey

Improvement	Secondary survey		Last survey	
	CVR	Mean士SD	CVR	Mean士SD
Introduction of an all-time practical test system	0.30	3.50±1.00	0.20	3.60±0.68
More rigorous rater training	0.70	4.20±0.70	0.90	4.30±0.57
Modification of the question development criteria	0.30	3.70±0.57	0.40	3.75±0.55
Adjustment of the number of questions among subjects	0.30	3.70±0.57	0.50	3.80±0.52
Introduction of problem-solving questions	0.50	4.00±0.72	0.70	4.10±0.64
Adjustment of difficulty of the national examination	0.60	4.00±0.65	0.80	4.05±0.51
Introduction of a specialized dental hygienist system	0.80	4.35±0.93	0.80	4.40±0.94
More rigorous refresher training for licensed dental hygienists	0.80	4.25±0.79	0.80	4.30±0.65

CVR, content validity ratio; SD, standard deviation.

## Supplementary materials

Supplement 1. Data file is available from https://dataverse.harvard.edu/dataset.xhtml?persistentId=doi%3A10.7910%2FDVN%2FHEJHYI

Supplement 2. Audio recording of the abstract.

## References

[1] Korean Dental Hygienists Association (2016). Definition of dental hygienist [Internet]. http://www.kdha.or.kr/introduce/dentalhygienist.aspx#introduce.

[2] Joint Commission on National Dental Examinations (2016). National Board Dental Hygiene Examination 2016 Guide [Internet]. http://www.ada.org/en/jcnde.

[3] National Dental Hygiene Certification Board (2017). National Dental Hygiene Certification Board Examination Candidate Guide [Internet]. https://www.ndhcb.ca/home-2.

[4] Kim SH (2008). Study on contents improvement of Korean Dental Hygienist National Examination.

[5] Christensen K, Doblhammer G, Rau R, Vaupel JW (2009). Ageing populations: the challenges ahead. Lancet.

[6] Theile CW, Strauss SM, Northridge ME, Birenz S (2016). The oral health care manager in a patient-centered health facility. J Evid Based Dent Pract.

[7] Kim SH, Jang JH, Oh SH (2009). A study for the improvement subjects of the Korean dental hygienists’ licensing examination. J Dent Hyg Sci.

[8] Korea Health Personnel Licensing Examination Institute (2007). Kuksiwon year book 2006.

[9] Korea Health Personnel Licensing Examination Institute (2015). Kuksiwon year book 2014.

[10] Nam YO, Ju UJ, Kim MJ (2013). A study on the improvement of the national dental hygienist practical examination. J Korean Soc Dent Hyg.

[11] Commission on Dental Accreditation (2016). Accreditation standard for dental hygiene education programs.

[12] The Canadian Dental Hygienists Association, The Canadian Dental Hygienists Association (2015). Core competencies. Canadian competencies for Baccalaureate Dental Hygiene Programs.

[13] Nam YO, Yoo JH (2014). Education, certification system and extent of duty in dental hygienists of developed countries. J Korean Soc Dent Hyg.

[14] American Dental Hygienists Association 100 year of dental hygiene [Internet]. http://www.tiki-toki.com/timeline/entry/55646/100-Years-of-Dental-Hygiene/.

