# Supplementary material for: Current issues and areas for improvement in the Korean Dental Hygienist National Licensing Examination: an expert Delphi survey among dental hygienists
Source: J Educ Eval Health Prof. 2017 Sep 13;14:21. doi: 10.3352/jeehp.2017.14.21 (PMC5676017; doi:10.3352/jeehp.2017.14.21)
Supplement: Supplementary file 2 [file jeehp-14-21-app1.pdf]

Appendix 1. First Delphi survey questionnaire

Q 1. What do you think about the issues facing the Korean Dental Hygienist National Licensing Examination? Please describe the issues in detail.

Response	

Q 2. Please write down your specific opinion on how to resolve the issues facing the Korean Dental Hygienist National Licensing Examination.

Response	

Q 3. What do you think are the core competencies of dental hygienists?

Response	

Q 4. Please write down your views on future developments in dental health.

Response	
